# Phases I–III Clinical Trials Using Adult Stem Cells

**DOI:** 10.4061/2010/579142

**Published:** 2010-11-04

**Authors:** Ricardo Sanz-Ruiz, Enrique Gutiérrez Ibañes, Adolfo Villa Arranz, María Eugenia Fernández Santos, Pedro L. Sánchez Fernández, Francisco Fernández-Avilés

**Affiliations:** Cardiology Department, Hospital General Universitario Gregorio Marañón, Doctor Esquerdo 46, 28007 Madrid, Spain

## Abstract

First randomized clinical trials have demonstrated that stem cell therapy can improve cardiac recovery after the acute phase of myocardial ischemia and in patients with chronic ischemic heart disease. Nevertheless, some trials have shown that conflicting results and uncertainties remain in the case of mechanisms of action and possible ways to improve clinical impact of stem cells in cardiac repair. In this paper we will examine the evidence available, analyze the main phase I and II randomized clinical trials and their limitations, discuss the key points in the design of future trials, and depict new directions of research in this fascinating field.

## 1. Concept and Types of Randomized Clinical Trials

A randomized clinical trial (RCT, also clinical study) is a research study in human volunteers to answer specific health questions. In other words, it is a rigorously controlled test of a new drug or a new invasive medical device on human subjects, in order to evaluate their effectiveness and safety by monitoring their effects on large groups of people. 

In the present state of clinical research, carefully conducted RCT are the fastest and safest ways to find treatments that work in people and ways to improve health. Interventional trials determine whether experimental treatments or new ways of using known therapies are safe and effective under controlled environments. Observational trials address health issues in large groups of people of populations in natural settings. 

All RCT must be conducted according to strict scientific and ethical principles. Every clinical trial must have a protocol, or action plan that describes what will be done in the study, how it will be conducted, and why each part of the study is necessary, including details such as the criteria for patient participation, the schedule of tests, procedures, and medications, and the length of the study.

RCT are conducted in a series of steps, called phases. Each phase is designed to answer a separate research question.

1. Phase I: researchers test a new drug or treatment in a small group of people for the first time to evaluate its safety, determine a safe dosage range, and identify side effects.
2. Phase II: the drug or treatment is given to a larger group of people to see if it is effective and to further evaluate its safety.
3. Phase III: the drug or treatment is given to large groups of people to confirm its effectiveness, monitor side effects, compare it to commonly used treatments, and collect information that will allow the drug or treatment to be used safely.
4. Phase IV: studies are done after the drug or treatment has been marketed to gather information on the drug's effect in various populations and any side effects associated with long-term use.

## 2. Clinical Research in Stem Cell Therapy: Same Methodology with a New Objective

Recent advances in reperfusion strategies have dramatically reduced early mortality after acute myocardial infarction (AMI), but as a result there is a higher incidence of heart failure among survivors. Optimal medical therapy and device implantation can improve the prognosis and the quality of life of these patients. Nevertheless, mortality and rehospitalization rates are still high and entail an overwhelming cost.

The field of cardiac cell therapy has emerged as a new alternative in this situation, and has made rapid progress. Its final goal is to repair the damaged myocardium and to restore cardiac function. Nevertheless, this is a real therapeutic challenge, given the facts that the loss of cardiomyocytes after an AMI is in the order of 1 billion cells, that supporting cells have to be supplied together with cardiomyocytes and that environmental signals which guide stem cells to the cardiac lineage or to the secretion of paracrine factors might be absent in such a damaged tissue [1]. 

Studies evaluating this new approach during the last 15 years have overall succeeded to a greater or lesser extent, and evidence available so far is encouraging. Phase I and II RCT indicate that cell therapy is a safe treatment which can improve cardiac function after AMI and in the chronic phase of coronary artery disease (CAD). Trial results are not uniform, however, probably due (1) to a lack of standardization and optimization of cell isolation and delivery protocols, (2) to a lack of a universally accepted nomenclature and imprecise use of terminology, and (3) to the large number of stem cell types under investigation in different clinical settings. These persisting mechanistic uncertainties about stem cell therapy should not preclude continuing clinical trials, which often provide the unique opportunity of identifying issues missed by our suboptimal preclinical models.

Moreover, these inconsistencies can be avoided or reduced if classical scientific methodology is followed. Although considered a relatively new field of research, stem cell experimentation must invariably walk on the path of the scientific method. Since Erasistratus of Chios in the third century before Christ and then after Aristotle's time, scientific method has been used as a way to ask and answer scientific questions by making observations and doing experiments. It includes a series of steps, that is, (1) asking a question, (2) doing background research, (3) constructing a hypothesis, (4) testing the hypothesis by doing an experiment, (5) analyzing the data and drawing a conclusion, and (6) communicating the results.

In the case of stem cell therapy, RCT started questioning if there was a possibility of repairing the heart after different types of tissular damage. Background evidence has already demonstrated that this possibility exists through stem cell administration in several preclinical models of cardiomyopathy. Thus, the key points in the design of present and future RCT in humans are (1) to formulate an adequate hypothesis, (2) to select the ideal population, cell type, and delivery method, and (3) to develop a correct and precise protocol. These decisions must be made in the light of previous evidence and with a translational mentality, in which experimental/preclinical data should help to design RCT, and, inversely, results of human studies should transfer new questions and hypothesis to the laboratory/bench side.

## 3. Clinical Scenarios in Stem Cell Therapy: Evidence Available

Stem cell therapy has accumulated growing evidence in different physiopathological conditions in small and large animal models, but human research has been almost limited to CAD. In this paper we will focus on *r* *a* *n* *d* *o* *m* *i* *z* *e* *d* placebo-controlled clinical trials in humans (Tables 1, 2, 3, and 4). Nonetheless, we will comment only on the largest and most relevant ones, as a way to analyze procedure-related variables that could have determined treatment outcomes and to address their limitations.

Natural history of CAD can be divided into acute (AMI) and chronic phases (chronic ischemic heart disease). In the latter stem cell therapy has been investigated in the subset of (1) ischemic heart failure (ventricular dysfunction) and (2) chronic myocardial ischemia (refractory angina).

In patients where restoration of contractile function is the clinical goal—such as those with end-stage ischemic heart failure or those early postinfarction—delivering cells with contractile potential may be of high priority. Under these conditions, naturally myogenic cells (i.e., skeletal myoblasts, cardiomyocytes, or any progenitor cell driven down a muscle lineage) appear to be a better first choice. However, on the one hand, formation of new myocardial mass has only been strictly established for embryonic stem cells (ESC), and is a process that has been achieved in very few trials and in small percentages with adult stem cells. And on the other hand, most of the studies after AMI have used bone marrow mononuclear cells (BMMCs) as an easily accessible source of adult stem and progenitor cells.

In conditions where chronic ischemia prevails, the angiogenic potential of the cells seems a more reasonable approach. In this case, BMMC, endothelial progenitor cells (EPCs), vascular progenitor cells or blood-derived multipotent adult progenitor cells and mesenchymal stem cells (MSCs) may be better choices than myogenic precursors.

### 3.1. Stem Cell Therapy after Acute Myocardial Infarction

Several trials have evaluated stem cell therapy after AMI, some with positive results and some with neutral ones. All of them used the intracoronary route, once the patency of the infarct-related artery was restored, and most of them with the mononucleated fraction of the bone marrow (Table 1). Four main RCT have been published with positive findings so far. In the BOOST trial [3], BMMC were proved to improve left ventricular (LV) contractility in the infarct border zone and global LV ejection fraction (LVEF) by 6%. However, only patients with larger infarcts showed maintained benefits in terms of LVEF at long followup (18 months). In the REPAIR-AMI trial [4], infusion of BMMC promoted an increase in LVEF of 2.8% at 12 months. The FINCELL trial [8] reported an improvement of 5% in LVEF after BMMC delivery. Finally, in the REGENT trial [12], patients treated with BMMC and with CXCR4^+^/CD34^+^ BMMC showed an increase of 3% in LVEF which was not observed in the control group, but these differences were not significant between treated and control patients at 6-month followup. This trial was limited by imbalances in baseline LVEF and by incomplete followup.

On the other hand, three RCTs resulted in neutral findings. Janssens et al. [5] reported no changes in LVEF after BMMC infusion, but a reduction in the infarct volume and an improvement in regional contractility in the greatest transmural infarct cases were observed in treated patients. In the ASTAMI trial [6] no significant effects on LVEF, LV volumes, or infarct size were observed after BMMC administration. The smaller number of cells and differences in the cell isolation protocol were invocated to explain these findings. Finally, in the HEBE trial [11], presented at the AHA Scientific Sessions in 2008, no changes in global or regional LV systolic function were reported after BMMC and mononucleated cells isolated from peripheral blood. 

So far, no safety concerns after BMMC intracoronary infusion have emerged. The risk of a higher rate of instent restenosis was not confirmed in the FINCELL trial [8] and in two recent meta-analyses [13, 14]. Moreover, none of the trials reported an increased incidence of malignant arrhythmias with BMMC [1].

Two trials have used MSC after AMI. The study by Chen et al. [2] demonstrated an improvement in LVEF and perfusion with intracoronary infusion of these cells, but these results have not been duplicated. Hare et al. [15] intravenously administered allogeneic MSC after an AMI with no higher rate of MACE and some benefits in terms of LVEF.

**Figure 1:**
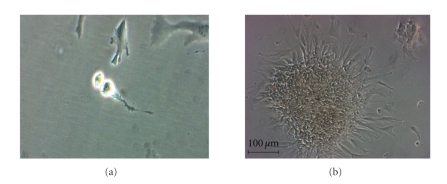
Autologous adipose-derived mesenchymal stem cells during mitosis (a) and growing in colonies in the 6th day of culture (magnification ×10, (b)). These cells were expanded from the adipose tissue stroma-vascular fraction under good manufacturing practice (GMP) conditions in our Cell Production Unit (Hospital Gregorio Marañón, Madrid).

New types of cells are also being explored, like adipose-derived stem cells (ADSCs) (Figure 1). No evidence is available to date, but the first-in-man RCT with intracoronary administration of freshly isolated ADSC after AMI (the APOLLO trial) has been recently completed. 

Another approach for stem cell therapy after AMI is cell mobilization from the bone marrow with the administration of granulocyte colony-stimulating factor (G-CSF). Several RCTs have been published, but results have been somehow less encouraging (Table 2). Only three trials have reported positive results. In the FIRSTLINE-AMI trial [16], the RIGENERA study [17], and in the study by Takano et al. [18], significant improvements in LVEF were observed. The rest of the trials showed negative findings.

Finally, the MAGIC trials used a combination of G-CSF and intracoronary injection of peripheral blood progenitor cells. In the first trial no differences in LVEF were noted, and an increase in instent restenosis rate was observed (G-CSF administration before bare-metal stent implantation) [19]. Then the investigators changed the design and used drug-eluting stents. In the MAGIC 3-DES trial, positive results in terms of LVEF were found after mobilization and intracoronary injection of isolated cells [20].

### 3.2. Stem Cell Therapy for Chronic Ischemic Heart Disease

#### 3.2.1. Ischemic Heart Failure

Skeletal myoblasts and BMMC have been used in heart failure (HF) patients (Table 3). The MAGIC trial [21], with transepicardial injection of SM during coronary artery bypass grafting (CABG) surgery, reported no changes in global or regional contractility. However, a reduction in LV end-diastolic and end-systolic volumes was observed in the high-dose group. Moreover, a trend towards a higher incidence of ventricular arrhythmias was noted. Dib et al. [22] reported an improvement in LVEF and viability after SM transendocardial injection, in contradiction with the SEISMIC trial (presented by Serruys at the 2008 ACC meeting) which showed no benefit of the same procedure at 6 months. 

In the TOPCARE-CHD trial [23], BMMC intracoronary delivery into the coronary artery supplying the most dyskinetic LV area showed an increase in LVEF of 2.9%, whereas progenitor circulating cells infusion and controls did not show any positive change. No major adverse cardiac events (MACE) were reported in this trial.

#### 3.2.2. Chronic Myocardial Ischemia

Patients with advanced CAD and no further options of revascularization (“no-option” patients) have also been studied in stem cell therapy trials (Table 4). Three RCT have been completed using the transendocardial route after electromechanical mapping of the LV, with BMMC or blood-derived progenitor cells. Losordo et al. [24] studied peripheral CD34^+^ cells isolated after G-CSF injections. Angina frequency and exercise time were improved, but no clear effects on myocardial perfusion were observed. In the PROTECT-CAD trial [25], BMMC injections improved NYHA functional class, exercise time, LVEF, wall thickening, and stress-induced perfusion defects. Finally, Van Ramshorst et al. [26] reported better LVEF, myocardial perfusion, angina functional class, exercise capacity and quality of life after BMMC administration. 

**Figure 2:**
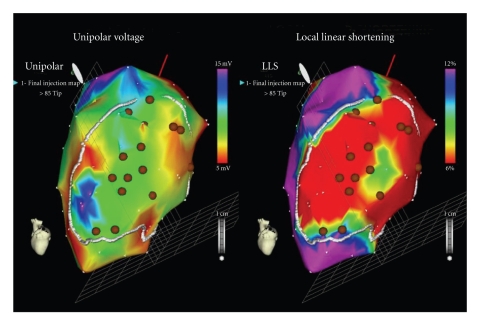
Electromechanical mapping of the left ventricle with the NOGA XP System (BDS, Cordis Corporation, Johnson and Johnson) from a patient enrolled in the PRECISE trial in our centre. Myocardial areas with low contractility and impaired endocardial voltage are identified as viable and targeted for cell injection (brown dots).

ADSC have also been studied in this type of patients. The PRECISE trial is a prospective, double blind, RCT that has randomised 27 patients with end-stage CAD not amenable for revascularization and with moderate-severe LV dysfunction to receive freshly isolated ADSC or placebo in a 3 : 1 ratio. The cells were delivered via transendocardial injections after LV electromechanical mapping with the NOGA XP delivery system (BDS, Cordis Corporation, Johnson and Johnson) (Figure 2), and results are still waiting for publication.

## 4. Key Issues for the Design of Future Stem Cell Therapy Trials

### 4.1. Ethical Considerations

As with any novel medical intervention, with stem cell therapy there is an ethical dichotomy between the need for new therapeutic approaches and rigorous scientific evidence regarding the safety and efficacy of the procedures. 

Due to its highly innovative nature, stem cell therapy should focus on reducing risks and providing rigorous evidence of efficacy and safety. Fundamental ethical requirements in this case include an acceptable balance of benefits and risks, informed and voluntary consent and equitable selection of subjects [27]. With transplantation of pluripotent cells (i.e., ESC and induced progenitor cells), additional safeguards are warranted because of the innovative nature of these treatments, differences between animal and human physiology, limited experience with these cells in humans, and the high hopes of desperate patients for whom no alternative effective treatment currently exists.

Some specific ethical recommendations have been given for RCT with stem cells [27]. They include the following.

1. Phase I-II trials should enroll participants in late stages of serious illness, such as persons with advanced or refractory disease, but not so ill that they are at greatly increased risk for adverse events.
2. Use a proper control group, in order to evaluate the positive effects of treatment and to ascribe culpability to any MACE seen with cell therapy. Then stem cells can be offered to the control group at the conclusion of the trial if the results show short-term benefit (“cross-over”).
3. Use clinically meaningful endpoints (see below, Section 4.5).
4. Coordinate scientific and ethical review, judging the potential clinical benefit of the treatment and assessing the scientific justification for the trial, including proof-of-principle and preclinical data on safety and dosage.
5. Verify that participants clearly understand the features of the trial. Since a comprehensive informed consent form may not prevent misconceptions about the trial, additional information should be given to those patients with significant misunderstandings. Participants should appreciate that researchers may not know whether or not the stem cell treatment will be beneficial, that animal studies might not predict effects of the cells in humans, and that unexpected adverse events may occur.
6. Ensure publication of results, even negative ones. For the interest of patients, researchers, and sponsors, negative findings cannot be withheld from publication.

### 4.2. Patient Selection and Delivery Methods

Patients with larger AMI or with severely depressed baseline LVEF and stroke volumes, or those with transmural extent of the infarct seem to benefit the most after BMMC treatment [1]. Conversely, patients with microvascular obstruction may not respond to intracoronary infusion of cells. Therefore, patient selection before conducting a RCT must take into account the pathophysiologic basis of the disease and baseline characteristics of the patients. For instance, it is well known that age, cardiovascular risk factors, and previous heart failure have a negative impact on the potentiality and functional capacity of the cells.

On the other hand, exploration of new delivery methods is mandatory, due to the low rate of cell retention, engraftment, and survival in the myocardium with the present routes of administration. New devices include transcoronary arterial injection into the perivascular space, improvements in transendocardial injection needle design, and the fusion of different imaging techniques for a more precise delivery (i.e., X-ray/MRI suites used in conjunction with electroanatomic maps of the LV).

### 4.3. Host Tissue and Cell-Related Issues

The two main determiners of cardiovascular repair are stem cells and injured myocardial tissue in which these cells are delivered. Both play the central role that will establish the efficacy of the treatment, and knowledge of the molecular/cellular changes and interactions between them is crucial when designing new RCT. 

After AMI, if blood flow is not restored quickly, cell death and myocardial necrosis are definitive. This activates a complement cascade with free radical and cytokine generation that recruits leukocytes and initiates the inflammatory response. Inflammation, while potentially detrimental to surviving cardiomyocytes, is necessary to clear away the debris (clearance of necrotic cells) and orchestrate downstream healing events. Chronic inflammatory cells such as macrophages and mast cells secrete cytokines and growth factors, which in turn activate fibroblasts to proliferate and synthesize collagen, a major component of the scar that replaces cardiomyocyte loss. Neovascularization is also stimulated by the release of growth factors from the inflammatory cells. Scar remodeling may continue for months to years, depending on the extent of the initial ischemic event [33]. 

LV remodeling, defined as post-AMI changes in wall structure, chamber geometry, and pump function, is mainly caused by changes in extracellular matrix (ECM). Cardiac ECM not only supports and aligns cardiomyocytes, thereby preserving a fundamental mechanical relationship by which sarcomeric shortening is translated to muscle force contraction, but also has signaling functions. Indeed, ECM is a storage depot for growth factors, hormones, and cytokines, and uses integrins to communicate with cells [33]. All these functions are lost after myocardial ischemia due to the release from inflammatory and endogenous cells of matrix metalloproteinases (MMP) and cytokines. MMP degrade ECM, disengage integrins, and stimulate reparative fibrosis. Cytokines like tumor necrosis factor *α* (TNF-*α*) and interleukins like IL-1 and IL-6 induce MMP synthesis and are related to the development of LV dysfunction, pulmonary edema, endothelial dysfunction, and cardiomyocyte apoptosis [34]. 

These cellular and signaling processes that constitute the proliferative phase of infarct healing in the myocardium influence and determine the fate of implanted stem cells. Ischemic myocardium constitutes an inflammatory hostile environment for stem cells, which is devoid of nutrients and oxygen and lacks survival signals from the ECM and cell-to-cell interactions. Indeed, only a small fraction of them survives in such adverse conditions. Nevertheless, some studies have shown that certain implanted stem cells may improve or counteract this situation. Intramyocardial transplantation of EPC after AMI induces significant and sustained increase in angiogenic, antiapoptotic, and chemoattractant factors, that are upregulated in both transplanted and host cells (i.e., vascular endothelial growth factor-A [VEGF-A], fibroblast growth factor-2 [FGF-2], angiopoietin-1 [Ang-1], angiopoietin-2 [Ang-2], placenta growth factor [PIGF], hepatocyte growth factor [HGF], insulin-like growth factor-1 [IGF-1], platelet-derived growth factor-B [PDGF-B], and stromal cell-derived factor-1 [SDF-1]) [35]. These humoral factors provide an additional favorable milieu for neovascularization and repair or regeneration of ischemic myocardium. Furthermore, there is a cross-talk between the heart and the bone marrow mediated by humoral effects that may improve this therapeutic effect: it has been proved that EPC transplantation further mobilizes endogenous BMMC into peripheral circulation, recruiting them into the ischemic myocardium [35].

Having these considerations in mind, new lines of research are being developed to improve cell survival rates in the ischemic myocardium, between them [1] are the following.

1. Preconditioning of the myocardium to retain a higher number of cells: low-energy shock waves, ultrasound-mediated destruction of microbubbles in the coronary circulation, and extracorporeal shock wave treatment have proved to increase retention of EPC, BMMC, and MSC. The last of these techniques is undergoing clinical testing in the Cellwave trial.
2. Activation or increase of chemotactic factors to attract cells to the damaged area: high mobility group box-1 (HMGB-1), SDF-1 or its receptor CXCR4, *β*_2_ integrin and endothelial nitric oxide synthase can be activated to increase the rate of homing of different types of stem cells (i.e., progenitor blood cells, EPC).

Regarding stem cells administered to the myocardium, their functional activity is determined by age and cardiovascular risk factors. As a consequence, future phase II-III RCT will explore cell enhancement strategies intended to increase their therapeutic potential. Several strategies are currently under investigation [1].

1. Pretreatment of the patients with drugs to stimulate cell potentiality: statins, rosiglitazone, and nitric oxide synthase enhancer AVE9488 can improve the migratory, invasive, and neovascularization capacity of EPC.
2. Strategies to prolong cell survival: between them, the use of a combination of growth factors to stimulate the expression of cardiomyocyte genes in MSC (currently under clinical investigation in the C-Cure trial), the use of heat shock to increase the resistance of cells to external stressors and the pretreatment of ESC-derived cardiomyocytes with heat shock and a cocktail of survival factors, are being explored.
3. Genetic modification of the cells prior to administration: overexpression of antiapoptotic genes or genetic manipulation to maintain cell's functionality (i.e., capacity to secrete paracrine mediators, to connect with host myocardium, or to differentiate into specialized cardiac cell types) can be achieved through genetic cell engineering.
4. Combined injection of cells and biomaterials: BMMC encapsulation within scaffolds (epicardial patches) or peptide nanofibers represents another strategy that needs further investigation.

### 4.4. Mechanisms of Action

**Figure 3:**
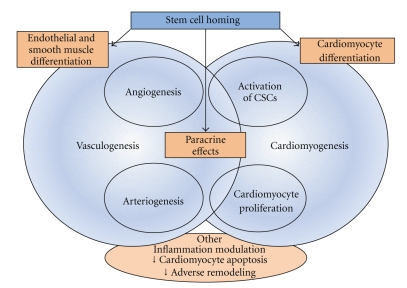
Proposed mechanisms of stem cell function after homing into the damaged heart. Note that differentiation processes and paracrine effects activate a cascade of events that interact actively to create new blood vessels and cardiomyocytes, with the final objective of functional cardiac repair. CSCs: cardiac stem cells.

Nowadays, it is believed that stem cell therapy could lead to successful cardiac regeneration or repair by any or a combination of three main general mechanisms (Figure 3): (1) differentiation of the administered cells into all of the cellular constituents of the heart (i.e., cardiomyogenesis and vasculogenesis processes), or, less probably, fusion of the administered cells with those, (2) release of factors capable of paracrine signaling from the administered cells, and (3) stimulation of endogenous repair by injected cells, through stem cell cardiac niches activation [36].

#### 4.4.1. Cardiomyogenesis and Vasculogenesis

While in the classic studies of the beginning of the decade (trans)differentiation of BMMC into cardiomyocytes, smooth muscle cells, and endothelial cells was postulated as the main mechanism that might explain the cardiac recovery resulting from stem cell therapy, this phenomenon has been demonstrated in low proportions in more recent studies. Regarding cellular fusion of administered cells with host myocardial ones, to date there is little evidence to support this hypothesis. 

#### 4.4.2. Paracrine Actions

Given that differentiation debate is still ongoing and that the number of newly generated cardiomyocytes and blood vessels is too low to explain significant functional improvements, the paracrine hypothesis is now considered the most plausible. According to this idea, the functional benefits of stem cells might be related to secretion of soluble factors that, acting in a paracrine fashion, protect the heart, attenuate pathological LV remodeling, induce neovascularization, and promote regeneration [37]. BMMC and MSC have been extensively studied and proved to produce and secrete a broad variety of cytokines, chemokines, and growth factors, between them VEGF, FGF, HGF, IGF, adrenomedullin, thymosin *β*4 (TB4), SDF-1, PDGF, and angiopoietin. These paracrine mediators are expressed/released in a temporal and spatial manner exerting different effects depending on the microenvironment after injury. In addition, these released factors may have autocrine actions on the biology of stem cells themselves [37].

The paracrine factors may influence adjacent cells and exert their actions via several mechanisms, including what follows.

1. Myocardial protection: MSC and BMMC in an ischemic environment release cytoprotective molecules that increase cardiomyocyte survival (VEGF, FGF, HGF, IGF-1, TB4, SDF-1, PDGF, and IL-1).
2. Neovascularization: BMMC, MSC, and EPC can give rise to vascular structures. The molecular processes leading to angiogenesis and arteriogenesis involve mediators such nitric oxide, VEGF, SDF-1, FGF, HGF, and angiopoietin.
3. Cardiac remodeling: paracrine factors released by transplanted stem cells may alter the ECM (i.e., inhibiting cardiac fibroblast proliferation and types I and III collagen synthesis), resulting in more favorable post-AMI remodeling and strengthening of the infarct scar. Stem cells (MSCs) may also produce molecules that limit local inflammation, thus reducing the remodeling process.
4. Cardiac contractility and metabolism: it has been demonstrated that stem cell therapy limits infarct size and prevents LV dysfunction. On the other hand, MSC and BMMC secrete inotropic factors (i.e., IGF-1) that positively modulate cell contractility, and these cells attenuate the profound bioenergetic abnormalities found in the border zone of myocardial infarction.
5. Cardiac regeneration: as we have seen, differentiation and cell fusion with native cardiomyocytes occur in very low rates after stem cell administration. Therefore, it is now believed that exogenous stem cell transplantation may activate resident cardiac stem cells (CSCs) and/or stimulate cardiomyocyte replication via paracrine action. Factors secreted by BMMC, MSC, and EPC, including HGF, SDF-1, VEGF, and IGF-1, enhance proliferation, mobilization, differentiation, survival, and function of CSC or even restoration of cardiac stem cell niches.

#### 4.4.3. Endogenous Repair

Finally, clonogenic and self-replicating endogenous CSCs have been isolated and cultured from human hearts. These CSCs—located in cardiac stem cell niches—have the capacity to differentiate into endothelial cells, smooth muscle myocytes, and cardiomyocytes. Though insufficient for a complete repair of the myocardium after any kind of insult, these cells can be activated by extracardiac delivered cells. Thus, administered allogeneic MSCs participate in maintaining stem cell niches, and through cell-to-cell interactions—apart from paracrine effects—may not only restore lost cellular constituents (differentiation) but also these niches with an ongoing and regulated self-replicating capacity [36].

### 4.5. Endpoints

Endpoints in RCT can be divided in hard and surrogate. The former include clinical endpoints such as survival, disease free survival or improvement in objective measures of disease-related functions, and are the ones that can change medical practice and present guidelines. The latter are defined as parameters or physical signs used as substitutes for an endpoint with clinical meaning that measures the quality of life or mortality [38]. In other words, they are intermediate markers (i.e., laboratory measurements) or response variables that can substitute for a “true” endpoint for the purpose of comparing specific interventions or treatments. Specifically, such response variables provide some additional information on true endpoint occurrence times for study subjects having censored values for such times. 

Hard or clinical endpoints have a direct relation with prognosis and survival. They include all-cause death, cardiac death, reinfarction, the need for further revascularizations, readmission due to heart failure and stroke, taken individually or as combined (composite) endpoints. Stem cell therapy will join the therapeutic spectrum for cardiovascular diseases only if it can be confirmed to improve quality of life and survival time with precise and accurate evidence of its influence on these parameters. Indeed, clinical variables have to be invariably the primary endpoints in large future phase III RCT.

The only trials that have explored the effect of stem cells in hard endpoints have been the REPAIR-AMI trial [4] and the BALANCE trial [39]. Both studies were not powered to detect differences in this kind of parameters (they were considered as secondary endpoints), and the latter was not even randomized. In the REPAIR-AMI trial the cumulative endpoint of death, recurrent AMI or necessity for revascularization was significantly reduced in the BMMC group compared with that in the placebo group after 12 months. Likewise, the combined endpoint of death, AMI and hospitalization for heart failure was also reduced. In the BALANCE study, BMMC transfer after AMI was associated with a significant reduction in mortality after 5 years of followup. Moreover, in two large meta-analyses [13, 14], a trend towards a beneficial effect of BMMC on death, reinfarction and rehospitalization for heart failure has also been reported. 

Having said that, surrogate endpoints have been a necessity with stem cell therapy RCT, because the sample size required to have statistical power to detect differences in mortality in these studies has been impractical [40]. For this reason, and although they have been used in phase I studies in order to do smaller and shorter trials, they should not be the primary endpoints in phase II-III RCT.

Parameters used as surrogate endpoints to assess the effects of stem cell therapy on survival in ischemic heart disease have to meet three requirements: (1) they must be closely correlated with survival, (2) changes in the parameters must reflect changes in the prognosis, and (3) there must be a pathophysiological basis to account for both relations [41]. Thus, there are several variables that have been used as surrogate endpoints, basically imaging (metabolism, perfusion, and contractility parameters) and laboratory measurements.

#### 4.5.1. Imaging Parameters

Many of the surrogate endpoints used in clinical research on heart disease are parameters obtained using imaging techniques, being LVEF the most frequently used. Since sample size depends on the standard deviation of the surrogate endpoint to be measured (which varies according to the variability of the imaging technique), the higher the spatial and temporal resolution of a given technique, the smaller the variability, especially if quantification is automatic [38]. Thus, techniques of choice to quantify LVEF in stem cell RCT are magnetic resonance imaging (MRI), single-photon emission computed tomography (SPECT), and contrast-enhanced echocardiography. The same techniques are the ideal ones for a proper evaluation of regional contractility (wall motion score index). Less accurate tools include simple echocardiography, computed tomography, and ventriculography.

However, if the study is conducted in patients who have had AMI, it is essential to calculate infarct size and thickness and thickening of the infarcted wall. For this purpose, MRI (through delayed-enhancement of gadolinium sequences) and SPECT are the best tools. If patients with depressed LVEF are included, we should use imaging techniques that appropriately measure LVEF and ventricular end-systolic and end-diastolic volumes (i.e., MRI and contrast echocardiography). 

Finally, if the study group consists of no-option patients, imaging techniques should be employed that appropriately assess myocardial perfusion (i.e., MRI and SPECT). In all stages of ischemic heart disease metabolism can be precisely studied by positron-emission tomography (PET) in combination with SPECT data, but MRI also offers relevant information.

Another more experimental surrogate parameters include the evaluation of microcirculation by gadolinium delayed-enhancement, inotropic responsiveness with stress (dobutamine) MRI, assessment of angiogenesis and arteriogenesis (first-pass of gadolinium), and new techniques of metabolic function, such as detection of high-energy phosphate metabolism and blood-oxygen tension determination using blood-oxygen-level-dependent (BOLD) MRI [40]. Again, in all these variables MRI plays a central role.

Nevertheless, despite all these robust imaging armamentarium, current stem cell therapies (RCTs) are still limited by the inability to perform imaging at the cellular level and track the fate of the cells (being radionuclide, MRI and reporter gene techniques the most used). Thus, the development of molecular imaging (with the use of MRI, PET, SPECT, computed tomography and echocardiography) and better techniques at the cellular level that allow 3-dimensional imaging will be necessary for clinical applications in the future [42]. Finally, it is clear that only RCT with hard clinical endpoints (i.e., cardiac death, reinfarction, rehospitalization, revascularization, and stroke) will definitively establish the role of stem cell therapy in ischemic heart disease. In this regard, we must not forget that imaging cannot provide a substitute for clinical outcome data.

#### 4.5.2. Laboratory Parameters

Several laboratory measurements have been used as surrogates in stem cell therapy RCT. Markers of inflammation, myocardial damage, and heart failure are the most frequently used. Between them, C-reactive protein (CRP), creatine-phosphokinase (CPK), T and I troponin, and probrain natriuretic peptide (pro-BNP) are universally available. Cytokines such as interleukins, and growth factors have also been used.

### 4.6. Followup

Another key point is final followup. Long-term followup should be carried out and specified in all RCT protocols in stem cell therapy, in order to precisely define the safety of the procedure (i.e., oncogenesis, restenosis, late adverse events).

### 4.7. The Consensus of the Task Force of the European Society of Cardiology for Future Trials: Next Directions

The Task Force of the European Society of Cardiology on stem cells and repair of the heart was created in 2006 to investigate and regulate the role of progenitor/stem cell therapy in the treatment of cardiovascular disease. It was almost four years ago that this group of experts and opinion leaders stated the type of studies needed [43].

1. Further large, double-blind, controlled RCT for the use of autologous BMMC in the treatment of AMI. The patient population should be all those presenting within 12 hours of AMI and treated with immediate revascularization, be it primary angioplasty or fibrinolysis.
2. Double-blind, controlled RCT for the use of autologous BMMC in the treatment of AMI in those patients presenting late (>12 hours) or who fail to respond to therapy (candidates for “rescue” angioplasty). Although, these groups may represent a small proportion of all patients with AMI, their prognosis remains poor.
3. Double-blind, controlled RCT for the use of autologous BMMC or SM in the treatment of ischemic heart failure. At some stage, the role of autologous stem/progenitor cells in the treatment of cardiomyopathies (in particular, dilated cardiomyopathy) will need to be examined.
4. A series of well-designed small studies to address safety or mechanism to test specific hypotheses (i.e., studies with labelled cells or to investigate paracrine or autocrine mechanisms). Such hypotheses would have arisen from basic science experiments.
5. Studies to confirm the risk/benefit ratio of the use of cytokines alone (i.e., G-CSF) or in conjunction with stem/progenitor cell therapy.

This Task Force also underlined the necessity for studies with hard clinical endpoints, MACE, subjective benefit and economic gain [43]. Another key point is standardization, both in outcome measures and in the processing of cells (better achieved in specialized centers following Good Manufacturing Procedure routines), in order to derive meaningful comparisons. Since these trials will need to include thousands of patients, they should be multicentre and ideally pan-European. An example of this new phase III large-scale trials is the RCT conducted by Zehier's group in Frankfurt, that will enroll up to 1200 patients with extensive AMI (LVEF <40%) and with a combined primary endpoint of cardiac death, reinfarction and rehospitalization due to heart failure. On the other hand, the Task Force stated that small uncontrolled trials with BMMC should be avoided, as they are unlikely to add anything new to the field.

Finally, next directions of cardiac cell therapy include what follows.

1. The study of the array of bioactive molecules that are secreted by stem cells, which have been demonstrated to induce neovascularization, modulate inflammation, fibrogenesis, cardiac metabolism, and contractility, increase cardiomyocyte proliferation and activate resident stem cells. The exhaustive analysis of this “secretomes” of BMMC, MSC, or EPC would lead to a better understating of the mechanisms of action of the cells and to a hypothetical protein-based therapy (off-the-shelf, noninvasive, systemic, and repetitive administration).
2. The use of different sources of pluripotent stem cells, like ESC, spermatogonial stem cells and oocytes. A new era has been initiated with the possibility of reprogramming adult cells (skin fibroblasts) to a pluripotent state by retroviral transduction [44, 45]. These “induced-pluripotent stem cells” (iPSs) show the characteristics of ESC and can differentiate to cardiomyocytes. New retroviral vectors and even nonviral vectors have been developed to reduce the risk of mutagenesis, and genetic modification of cells with suicide genes have been proposed to reduce the risk of tumor formation.
3. The creation of bioartificial hearts after a process of decelularization with detergents, obtention of the underlying extracellular matrix (cardiac architecture), and stem cell repopulation [46]. The “acellular” heart can then be reseeded with cardiac stem cells or EPC, showing contractile activity in animal models. This new approach of tissue bioengineering has opened a fascinating era in cardiovascular medicine.

## 5. Conclusions

Although mixed results have emerged from the first stem cell therapy RCT in cardiovascular medicine, the overall data suggest that these procedures are feasible and safe in both acute and chronic scenarios of ischemic heart disease. After phase I-II RCT, it is clear that BMMC transfer after AMI has the potential to improve the recovery of LV systolic function beyond what can be achieved by current interventional and medical therapies. In chronic ischemic heart disease, SM and BMMC have proved to improve myocardial perfusion and contractile performance.

New types of cells (including ADSC and iPS), improvements in delivery and imaging methods, strategies to enhance cell potentiality or to improve the myocardial proinflammatory microenvironment, and the creation of bioartificial hearts are the main new directions of research in the near future. 

Finally, large-scale, phase III, double-blind, controlled RCT performed under rigorous safety standards are being initiated to prove unequivocal clinical benefits, including improved survival. These trials will definitively establish the effectiveness of stem cell therapy in improving clinical outcomes, confirming the real potential of cardiac regenerative therapy.

##  Conflicts of Interest 

None of the authors has conflicts of interest to declare.

## Figures and Tables

**Table 1:** Randomized clinical trials with stem cells in patients with acute myocardial infarction (intracoronary delivery).

Trial (year)	*n*	Cell type	Cell count	Days after AMI	Primary endpoint (followup)	Comments
Chen [2] (2004)	69	MSC	9 × 10^9^	18	Improved LVEF at 6 m	LVEF by echocardiography
BOOST [3] (2004)	60	BMMC	2 × 10^9^	6 ± 1	Improved LVEF at 6 m	Effect diminished after 18/61 m
REPAIR-AMI [4] (2006)	187	BMMC	2 × 10^8^	3–6	Improved LVEF at 4 m	LVEF by ventriculography
Janssens [5] (2006)	66	BMMC	2 × 10^8^	1	No change LVEF at 4 m	Improved regional contractility and reduction in infarct size
ASTAMI [6] (2006)	97	BMMC	7 × 10^7^	6 ± 1	No change LVEF at 6 m	LVEF ↑8% by SPECT, ↑1% by MRI
TCT-STAMI [7] (2006)	20	BMMC	4 × 10^7^	1	Improved LVEF at 6 m	LVEF by echocardiography
FINCELL [8] (2008)	77	BMMC	4 × 10^8^	3	Improved LVEF at 6 m	LVEF by ventriculography
Meluzin [9] (2006)	66	BMMC	1 × 10^7^ (low d)1 × 10^8^ (high d)	7	Improved LVEF at 3 m in high dose group	LVEF by SPECT
Penicka [10] (2007)	27	BMMC	3 × 10^9^	9	No change LVEF at 4 m	LVEF by echocardiography
HEBE [11] (2008)	189	BMMC versus PBC	—	3–8	No changes in global or regional LV function	Final results pending
REGENT [12] (2009)	117	BMMC (unselected, CD34^+^/CXCR4^+^)	2 × 10^8^ (unsel), 2 × 10^6^ (CD34^+^)	3–12	Improved LVEF with both cell types	LVEF by MRI (in 117/200 patients)

MSC: mesenchymal stem cells (bone marrow origin); BMMC: bone marrow mononuclear cells; PBC: peripheral blood cells; LVEF: left ventricular ejection fraction; LV: left ventricle; SPECT: single-photon emission computed tomography; MRI: magnetic resonance imaging.

**Table 2:** Randomized clinical trials with granulocyte colony-stimulating factor in patients with acute myocardial infarction (subcutaneous).

Trial (year)	*n*	Dose	Timing after AMI (PCI)	Followup	Comments
Valgimigli [28] (2005)	20	5 *μ*g/kg × 4 d	1 d	No change LVEF at 6 m	LVEF by SPECT
FIRSTLINE-AMI [16] (2005)	50	10 *μ*g/kg × 6 d	90 min	Improved LVEF at 4 m	LVEF by echocardiography
REVIVAL-2 [29] (2006)	114	10 *μ*g/kg × 5 d	5 d	No change LVEF at 5 m	LVEF by MRI
STEMMI [30] (2006)	78	10 *μ*g/kg × 6 d	28 h	No change LVEF at 6 m	LVEF by echocardiography and MRI
G-CSF-STEMI [31] (2006)	44	10 *μ*g/kg × 5 d	35 h	No change LVEF at 3 m	LVEF by MRI
Ellis [32] (2006)	18	5 *μ*g/kg × 5 d (low d), 10 *μ*g/kg × 5 d (high d)	<30 h	Improved LVEF at 30 d	LVEF by echocardiography
RIGENERA [17] (2007)	41	10 *μ*g/kg × 5 d	5 d	Improved LVEF at 6 m	LVEF by echocardiography
Takano [18] (2007)	40	2,5 *μ*g/kg × 5 d	1 d	Improved LVEF at 6 m	LVEF by SPECT
MAGIC [19] (2004)*	27	10 *μ*g/kg × 4 d; PBC: 1 × 10^9^	1 d	No change LVEF at 6 m	LVEF by SPECT
MAGIC 3-DES [20] (2006)*	50	10 *μ*g/kg × 3 d; PBC: 2 × 10^9^	1 d	Improved LVEF at 6 m	LVEF by MRI

*MAGIC trials used a combination of indirect mobilization (G-CSF) and direct intracoronary injection of peripheral blood cells (PBC); LVEF: left ventricular ejection fraction; SPECT: single-photon emission computed tomography; MRI: magnetic resonance imaging.

**Table 3:** Randomized clinical trials in patients with chronic ischemic heart failure.

Trial	*N*	Cell type	Delivery	Timing	Primary endpoint	Comments
MAGIC [21]	97	SM	transepi	>4 weeks	No change LVEF	Reduction in LVEDV/LVESV
Dib [22]	23	SM	transendo	>10 years	Improved LVEF and viability	—
SEISMIC	47	SM	transendo	chronic	No change LVEF	—
TOPCARE-CHD [23]	58	BMMC versus CPC	ic	81 ± 72 months	Improved LVEF w/BMMC	—

SM: skeletal myoblasts; BMMC: bone marrow mononuclear cells; CPC: circulating progenitor cells; transepi: tranepicardial; transendo: transendocardial; ic: intracoronary; LVEF: left ventricular ejection fraction; LVEDV: left ventricular end-diastolic volume; LVESV: left ventricular end-systolic volume.

**Table 4:** Randomized clinical trials in patients with chronic myocardial ischemia.

Trial	*N*	Cell type	Delivery	Timing	Primary endpoint	Comments
Losordo [24]	24	CD34^+^	transendo	chronic	Improved angina parameters	No clear perfusion benefit
PROTECT-CAD [25]	28	BMMC	transendo	chronic	Improved angina parameters	Improved LVEF and perfusion
Van Ramshorst [26]	50	BMMC	transendo	chronic	Improved angina parameters	Improved LVEF and perfusion

BMMC: bone marrow mononuclear cells; LVEF: left ventricular ejection fraction.

## References

[1] Wollert KC, Drexler H (2010). Cell therapy for the treatment of coronary heart disease: a critical appraisal. *Nature Reviews Cardiology*.

[2] Chen S-L, Fang W-W, Ye F (2004). Effect on left ventricular function of intracoronary transplantation of autologous bone marrow mesenchymal stem cell in patients with acute myocardial infarction. *American Journal of Cardiology*.

[3] Wollert KC, Meyer GP, Lotz J (2004). Intracoronary autologous bone-marrow cell transfer after myocardial infarction: the BOOST randomised controlled clinical trial. *Lancet*.

[4] Schächinger V, Erbs S, Elsässer A (2006). Intracoronary bone marrow-derived progenitor cells in acute myocardial infarction. *New England Journal of Medicine*.

[5] Janssens S, Dubois C, Bogaert J (2006). Autologous bone marrow-derived stem-cell transfer in patients with ST-segment elevation myocardial infarction: double-blind, randomised controlled trial. *Lancet*.

[6] Lunde K, Solheim S, Aakhus S (2006). Intracoronary injection of mononuclear bone marrow cells in acute myocardial infarction. *New England Journal of Medicine*.

[7] Ge J, Li Y, Qian J (2006). Efficacy of emergent transcatheter transplantation of stem cells for treatment of acute myocardial infarction (TCT-STAMI). *Heart*.

[8] Huikuri HV, Kervinen K, Niemelä M (2008). Effects of intracoronary injection of mononuclear bone marrow cells on left ventricular function, arrhythmia risk profile, and restenosis after thrombolytic therapy of acute myocardial infarction. *European Heart Journal*.

[9] Meluzín J, Mayer J, Groch L (2006). Autologous transplantation of mononuclear bone marrow cells in patients with acute myocardial infarction: the effect of the dose of transplanted cells on myocardial function. *American Heart Journal*.

[10] Penicka M, Horak J, Kobylka P (2007). IIntracoronary injection of autologous bone marrow-derived mononuclear cells in patients with large anterior acute myocardial infarction: a prematurely terminated randomized study. *Journal of the American College of Cardiology*.

[11] Van Der Laan AM, Hirsch A, Nijveldt R (2008). Bone marrow cell therapy after acute myocardial infarction: the HEBE trial in perspective, first results. *Netherlands Heart Journal*.

[12] Tendera M, Wojakowski W, Ruzyllo W (2009). Intracoronary infusion of bone marrow-derived selected CD34^+^CXCR4^+^ cells and non-selected mononuclear cells in patients with acute STEMI and reduced left ventricular ejection fraction: results of randomized, multicentre Myocardial Regeneration by Intracoronary Infusion of Selected Population of Stem Cells in Acute Myocardial Infarction (REGENT) Trial. *European Heart Journal*.

[13] Lipinski MJ, Biondi-Zoccai GGL, Abbate A (2007). Impact of intracoronary cell therapy on left ventricular function in the setting of acute myocardial infarction. A collaborative systematic review and meta-analysis of controlled clinical trials. *Journal of the American College of Cardiology*.

[14] Martin-Rendon E, Brunskill SJ, Hyde CJ, Stanworth SJ, Mathur A, Watt SM (2008). Autologous bone marrow stem cells to treat acute myocardial infarction: a systematic review. *European Heart Journal*.

[15] Hare JM, Traverse JH, Henry TD (2009). A randomized, double-blind, placebo-controlled, dose-escalation study of intravenous adult human mesenchymal stem cells (prochymal) after acute myocardial infarction. *Journal of the American College of Cardiology*.

[16] Ince H, Petzsch M, Kleine HD (2005). Preservation from left ventricular remodeling by front-integrated revascularization and stem cell liberation in evolving acute myocardial infarction by use of granulocyte-colony-stimulating factor (FIRSTLINE-AMI). *Circulation*.

[17] Leone AM, Galiuto L, Garramone B (2007). Usefulness of granulocyte colony-stimulating factor in patients with a large anterior wall acute myocardial infarction to prevent left ventricular remodeling (the rigenera study). *American Journal of Cardiology*.

[18] Takano H, Hasegawa H, Kuwabara Y (2007). Feasibility and safety of granulocyte colony-stimulating factor treatment in patients with acute myocardial infarction. *International Journal of Cardiology*.

[19] Kang H-J, Kim H-S, Zhang S-Y (2004). Effects of intracoronary infusion of peripheral blood stem-cells mobilised with granulocyte-colony stimulating factor on left ventricular systolic function and restenosis after coronary stenting in myocardial infarction: the MAGIC cell randomised clinical trial. *Lancet*.

[20] Kang H-J, Lee H-Y, Na S-H (2006). Differential effect of intracoronary infusion of mobilized peripheral blood stem cells by granulocyte colony-stimulating factor on left ventricular function and remodeling in patients with acute myocardial infarction versus old myocardial infarction: the MAGIC cell-3-DES randomized, controlled trial. *Circulation*.

[21] Menasché P, Alfieri O, Janssens S (2008). The myoblast autologous grafting in ischemic cardiomyopathy (MAGIC) trial: first randomized placebo-controlled study of myoblast transplantation. *Circulation*.

[22] Dib N, Michler RE, Pagani FD (2005). Safety and feasibility of autologous myoblast transplantation in patients with ischemic cardiomyopathy: four-year follow-up. *Circulation*.

[23] Assmus B, Honold J, Schächinger V (2006). Transcoronary transplantation of progenitor cells after myocardial infarction. *New England Journal of Medicine*.

[24] Losordo DW, Schatz RA, White CJ (2007). Intramyocardial transplantation of autologous CD34^+^ stem cells for intractable angina: a phase I/IIa double-blind, randomized controlled trial. *Circulation*.

[25] Tse H-F, Thambar S, Kwong Y-L (2007). Prospective randomized trial of direct endomyocardial implantation of bone marrow cells for treatment of severe coronary artery diseases (PROTECT-CAD trial). *European Heart Journal*.

[26] Van Ramshorst J, Bax JJ, Beeres SLMA (2009). Intramyocardial bone marrow cell injection for chronic myocardial ischemia: a randomized controlled trial. *Journal of the American Medical Association*.

[27] Lo B, Kriegstein A, Grady D (2008). Clinical trials in stem cell transplantation: guidelines for scientific and ethical review. *Clinical Trials*.

[28] Valgimigli M, Rigolin GM, Cittanti C (2005). Use of granulocyte-colony stimulating factor during acute myocardial infarction to enhance bone marrow stem cell mobilization in humans: clinical and angiographic safety profile. *European Heart Journal*.

[29] Zohlnhöfer D, Ott I, Mehilli J (2006). Stem cell mobilization by granulocyte colony-stimulating factor in patients with acute myocardial infarction: a randomized controlled trial. *Journal of the American Medical Association*.

[30] Ripa RS, Jørgensen E, Wang Y (2006). Stem cell mobilization induced by subcutaneous granulocyte-colony stimulating factor to improve cardiac regeneration after acute ST-elevation myocardial infarction: result of the double-blind, randomized, placebo-controlled stem cells in myocardial infarction (STEMMI) trial. *Circulation*.

[31] Engelmann MG, Theiss HD, Hennig-Theiss C (2006). Autologous bone marrow stem cell mobilization induced by granulocyte colony-stimulating factor after subacute ST-segment elevation myocardial infarction undergoing late revascularization: final results from the G-CSF-STEMI (Granulocyte Colony-Stimulating Factor ST-Segment Elevation Myocardial Infarction) trial. *Journal of the American College of Cardiology*.

[32] Ellis SG, Penn MS, Bolwell B (2006). Granulocyte colony stimulating factor in patients with large acute myocardial infarction: results of a pilot dose-escalation randomized trial. *American Heart Journal*.

[33] Lindsey ML, Mann DL, Entman ML, Spinale FG (2003). Extracellular matrix remodeling following myocardial injury. *Annals of Medicine*.

[34] Dewald O, Ren G, Duerr GD (2004). Of mice and dogs: species-specific differences in the inflammatory response following myocardial infarction. *American Journal of Pathology*.

[35] Cho H-J, Lee N, Ji YL (2007). Role of host tissues for sustained humoral effects after endothelial progenitor cell transplantation into the ischemic heart. *Journal of Experimental Medicine*.

[36] Mazhari R, Hare JM (2007). Mechanisms of action of mesenchymal stem cells in cardiac repair: potential influences on the cardiac stem cell niche. *Nature Clinical Practice Cardiovascular Medicine*.

[37] Gnecchi M, Zhang Z, Ni A, Dzau VJ (2008). Paracrine mechanisms in adult stem cell signaling and therapy. *Circulation Research*.

[38] San Román JA, Candell-Riera J, Arnold R (2009). Quantitative analysis of left ventricular function as a tool in clinical research. Theoretical basis and methodology. *Revista Espanola de Cardiologia*.

[39] Yousef M, Schannwell CM, Köstering M, Zeus T, Brehm M, Strauer BE (2009). The BALANCE Study: clinical benefit and long-term outcome after intracoronary autologous bone marrow cell transplantation in patients with acute myocardial infarction. *Journal of the American College of Cardiology*.

[40] Fuster V, Sanz J, Viles-Gonzalez JF, Rajagopalan S (2006). The utility of magnetic resonance imaging in cardiac tissue regeneration trials. *Nature Clinical Practice Cardiovascular Medicine*.

[41] San Román JA, Fernández-Avilés F (2006). The role of noninvasive imaging techniques in the assessment of stem cell therapy after acute myocardial infarction. *Nature Clinical Practice Cardiovascular Medicine*.

[42] George JC (2010). Stem cell therapy in acute myocardial infarction: a review of clinical trials. *Translational Research*.

[43] Bartunek J, Dimmeler S, Drexler H (2006). The consensus of the task force of the European Society of Cardiology concerning the clinical investigation of the use of autologous adult stem cells for repair of the heart. *European Heart Journal*.

[44] Takahashi K, Tanabe K, Ohnuki M (2007). Induction of pluripotent stem cells from adult human fibroblasts by defined factors. *Cell*.

[45] Yu J, Vodyanik MA, Smuga-Otto K (2007). Induced pluripotent stem cell lines derived from human somatic cells. *Science*.

[46] Ott HC, Matthiesen TS, Goh S-K (2008). Perfusion-decellularized matrix: using nature’s platform to engineer a bioartificial heart. *Nature Medicine*.

